# Long-term outcomes and prognostic factors in elderly patients with breast cancer: single-institutional experience

**DOI:** 10.3332/ecancer.2023.1542

**Published:** 2023-05-02

**Authors:** Tabassum Wadasadawala, Roshankumar Patil, Johnny Carlton, Shalini Verma, Namita Umesh, Pallavi Rane, Rajiv Sarin, Rima Pathak, Jyoti Bajpai, Nita Nair, Tanuja Shet, Ramneet Kaur

**Affiliations:** 1 Department of Radiation Oncology, Tata Memorial Centre, Homi Bhabha National Institute, Mumbai, Maharashtra 400012, India; 2 Department of Radiation Oncology, Tata Memorial Centre, Homi Bhabha Cancer Hospital, Varanasi, Uttar Pradesh 221010, India; 3 Department of Statistics, Tata Memorial Centre, Homi Bhabha National Institute, Mumbai, Maharashtra 400012, India; 4 Department of Medical Oncology, Tata Memorial Centre, Homi Bhabha National Institute, Mumbai, Maharashtra 400012, India; 5 Department of Surgical Oncology, Tata Memorial Centre, Homi Bhabha National Institute, Mumbai, Maharashtra 400012, India; 6 Department of Pathology, Tata Memorial Centre, Homi Bhabha National Institute, Mumbai, Maharashtra 400012, India; 7 Department of Radiation Oncology, Cancer Centers of America, Nashik, Maharashtra 422011, India; a https://orcid.org/0000-0003-2167-420X; b https://orcid.org/0000-0002-9025-6864

**Keywords:** elderly population, breast cancer, survival outcomes

## Abstract

**Introduction:**

Despite advances in treatment, there is rising mortality in elderly patients with breast cancer. We aimed to conduct an audit of non-metastatic elderly breast cancer patients to understand the predictors of outcome.

**Methods:**

Data collection was done from electronic medical records. All time-to-event outcomes were analysed using Kaplan–Meier method and compared using log-rank test. Univariate and multi-variate analysis of known prognostic factors was also done. Any p-value ≤0.05 was considered statistically significant.

**Results:**

A total of 385 elderly (>70 years) breast cancer patients (range 70–95 years) were treated at our hospital from January 2013 to December 2016. The hormone receptor was positive in 284 (73.8%) patients; 69 (17.9%) patients had over-expression of HER2-neu, while 70 (18.2%) patients had triple-negative breast cancer. A large majority of women (N = 328, 85.9%) underwent mastectomy while only 54 (14.1%) had breast conservation surgery. Out of 134 patients who received chemotherapy, 111 patients received adjuvant, while the remaining 23 patients received neoadjuvant chemotherapy. Only 15 (21.7%) patients of the 69 HER2-neu receptor-positive patients received adjuvant trastuzumab. Adjuvant radiation was given to 194 (50.3%) women based on the type of surgery and disease stage. Adjuvant hormone therapy was planned using letrozole in 158 (55.6%) patients, while tamoxifen was prescribed in 126 (44.4%). At the median follow up of 71.7 months, the 5-year overall survival, relapse-free survival, locoregional relapse-free survival, distant disease-free survival, breast cancer-specific survival were 75.3%, 74.2%, 84.8%, 76.1% and 84.5%. Age, tumour size, presence of lymphovascular invasion (LVSI) and molecular subtype emerged as independent predictors of survival on multi-variate analysis.

**Conclusion:**

The audit highlights the underutilisation of breast-conserving therapy and systemic therapy in the elderly. Increasing age and tumour size, presence of LVSI and molecular subtype were found to be strong predictors of outcome. The findings from this study will help to improve the current gaps in the management of breast cancer among the elderly.

## Introduction

Breast cancer is the leading cancer site in Indian women, accounting for 162,000 incident cases and 87,000 deaths annually [1]. The risk of developing breast cancer increases with advancing age [2]. The improving life expectancy in India is expected to increase the incidence of breast cancer to 235,000 by the year 2026 [3].

Despite advances in treatment, there is rising mortality in elderly patients with breast cancer [4]. This may be due to the inability to receive standard treatment (surgery, chemotherapy, or adjuvant radiotherapy (RT)) because of fear of advanced age and associated comorbidities. The older adult population has the competing risk of mortality from non-oncological causes due to increased prevalence of co-morbidities, which also impacts the rate of receiving standard cancer therapies. Thereby undertreatment can result in higher rates of recurrence and breast cancer-specific mortality [5]. Moreover, elderly patients with cancer are usually under-represented in clinical trials, because of varied reasons – increased co-morbidities, use of concomitant medications, relatively long-term endpoints chosen for clinical trials and poor family/social support leading to concerns of loss to follow up [6]. It has also been seen that elderly patients more often present in advanced stages at diagnosis. Theoretically, breast cancers in elderly patients have lower proliferation rates and are more likely to have favourable histology as well as molecular profile. However, an interplay of late diagnosis, disease biology and treatment interventions seem to impact breast cancer-specific mortality which is seen to be rising with advancing age [7].

Studies from Jordan [8] and Korea [9] have been reported earlier but there is overall a paucity of data regarding the clinical outcome of breast cancer in elderly from developing countries. We undertook this analysis intending to evaluate disease-specific survival outcomes and the factors affecting them.

## Methods and materials

After approval from our institutional ethics committee, non-metastatic elderly patients (≥70 years of age) with histologically proven breast cancer treated at our hospital from January 2013 to December 2016 were identified from a prospectively maintained breast-oncology database. Data regarding demography, clinical presentation, histopathological features, molecular profiling, treatment details and outcomes were retrieved from hospital case files and electronic medical records. A total of 385 consecutive cases identified from the database formed the study population for this retrospective analysis.

The primary objective was to assess overall survival (OS). Secondary objectives included reporting disease characteristics, incidence of co-morbidities, treatment characteristics, compliance too treatment and factors influencing the survival outcomes. Time-to-event endpoints were calculated as per Definitions for the Assessment of Time-to-event Endpoints in CANcer trials [10, 11]. OS was defined as the time from the date of diagnosis to the date of death from any cause or to the last date of follow-up. Relapse-free survival (RFS) was defined as the time from diagnosis to recurrence or date of death from any cause. Locoregional RFS (LRFS) was defined as the time from diagnosis to local or regional recurrence or date of death from any cause. Distant disease-free survival (DDFS) was defined as the time from diagnosis to distant recurrence or date of death from any cause. Breast cancer-specific survival (BCSS) was defined as the time from diagnosis to the date of death due to breast cancer.

Survival data were analysed using the Kaplan–Meier method and survival curves were compared by use of the log-rank test on univariate analysis. We have taken the average value for the null entries during univariate analysis of Karnofsky Performance Scale (KPS) to account for the missing data. In multivariable analyses, a Cox proportional hazards model was used to adjust for covariates of statistical significance. Any *p*-value ≤0.05 was considered statistically significant. Statistical analysis was performed using Statistical Package for Social Sciences (SPSS, Chicago, IL, USA) software version 21.

## Results

### Study cohort

An electronic search of the breast-oncology database identified a total of 385 newly diagnosed elderly (>70 years of age) breast cancer patients that were registered at our institute between January 2013 and December 2016.

### Baseline characteristics of the study participants

The median age in our population was 74 years (range 70–95) with 329 (85.4%) patients in the age group of 70–80 years, while 56 (14.6%) patients were above 80 years. Co-morbidities were seen in 253 (65.7%) patients. Of these, 67 (27.3%) patients had multiple co-morbidities and 184 (72.7%) patients had single comorbidity. The median pathological tumour size was 3 cm (Range: 0–8 cm) while lymph nodes were positive in 167 (43.3%) patients. Lymphovascular invasion (LVSI) was present in one-third (35%) while tumours were of high grade in two-thirds (64.6%) of the study population. Predominantly patients (73.8%) were hormone positive (either oestrogen receptor (ER) or progesterone receptor (PR) or both) and of luminal A or B subtype, followed by 70 (18.2%) and 31 (8.3%) patients each in triple-negative breast cancer (TNBC) and HER2 enriched subtypes. The patients with no comorbidities presented with higher T size as compared to patients with comorbidities (83% versus 75%; *p*: 0.09). Relevant baseline patient, disease-related characteristics of the study cohort are described in Table 1. The eighth edition of the American Joint Committee on Cancer (AJCC) staging system was followed for clinical staging.

### Treatment details

Three hundred and twenty-eight (85.9%) patients underwent mastectomy while only 54 (14.1%) underwent breast conservation surgery (BCS). Three patients did not undergo surgery despite advising the same. Twenty-three patients received neoadjuvant chemotherapy while, 111 patients received adjuvant chemotherapy. Out of these patients, 13 patients had grade 3 or higher toxicity in the form of febrile neutropenia in 7, peripheral neuropathy in 4 and cardiac toxicity in 2 patients. Only 15 (21.7%) patients of the 69 HER2-neu receptor-positive patients received adjuvant Trastuzumab. No other anti-HER2 therapy was advised. Adjuvant hormone therapy was planned using letrozole in 158 (55.6%) patients, while tamoxifen was prescribed in 126 (44.4%). Adjuvant radiation was planned for 194 (50.3%) women based on the type of surgery and disease stage. Of the 54 patients who underwent BCS, 13 patients did not receive adjuvant RT due to competing risk from multiple co-morbidities or observation was offered due to favourable disease profile. Post-mastectomy RT (PMRT) was advised for node-positive disease or locally advanced tumours at the time of presentation as per institutional protocol. The median dose fractionation of RT to breast or chest wall was 40 Gy in 15 fractions over a period of 3 weeks, while the median dose for the boost in the case of BCS was 12.5 Gy in 5 fractions. RT was tolerated well by most of the patients who had grade I acute skin reactions (86.9%) and grade I acute dysphagia (93.1%). Compliance towards the treatment is defined as the proportion of patients those who had finished the planned treatment completely. The overall compliance towards planned treatment was shown by 343 (89.9%) of patients. The compliance of patients towards surgery, radiation therapy and chemotherapy was 99.2%, 90.14% and 81.20%, respectively. The major reasons for non-compliance for the whole treatment completion included comorbidities (45.2% of patients had comorbidities; *p*: 0.003) and illiteracy (57.1% were illiterate; *p* < 0.001). The difference between compliance towards systemic treatment in patients with or without comorbidities was not significant (26% versus 34%; *p*: 0.06). Relevant treatment-related details of the study cohort are described in Table 1.

### Clinical outcomes

By the time of this analysis, 83 patients (21.5%) patients had died (52 to disease progression and 31 to other causes), 295 patients (76.6%) patients were documented alive (289 without disease and 6 with disease), while 7 patients (1.9%) were lost to follow-up. A total of 58 (15%) recurrences were seen in the entire cohort, out of which majority of them 34 (58.6%), had distance recurrences, while 13 (22.4%) had locoregional recurrences. Eleven patients had both local as well as distant recurrences. Out of these 58 patients with recurrences, only 4 (6.9%) were treated with radical intent and the remaining 54 (93.1%) were treated with palliative intent only. All four patients who were treated radically had presented with isolated local recurrence. All of them were hormone positive and had developed recurrence after a median disease-free interval of 18 months (range: 10–24 months). The most common salvage treatment was a combination of chemotherapy and RT for 11 (19%) patients followed by chemotherapy or RT alone for 6 (10.3%) patients. Twenty-five (43.1%) patients were not fit for any further treatment and were referred to palliative services for the best supportive care, while 12 (20.7%) patients defaulted for any further treatment.

**Figure 1.**
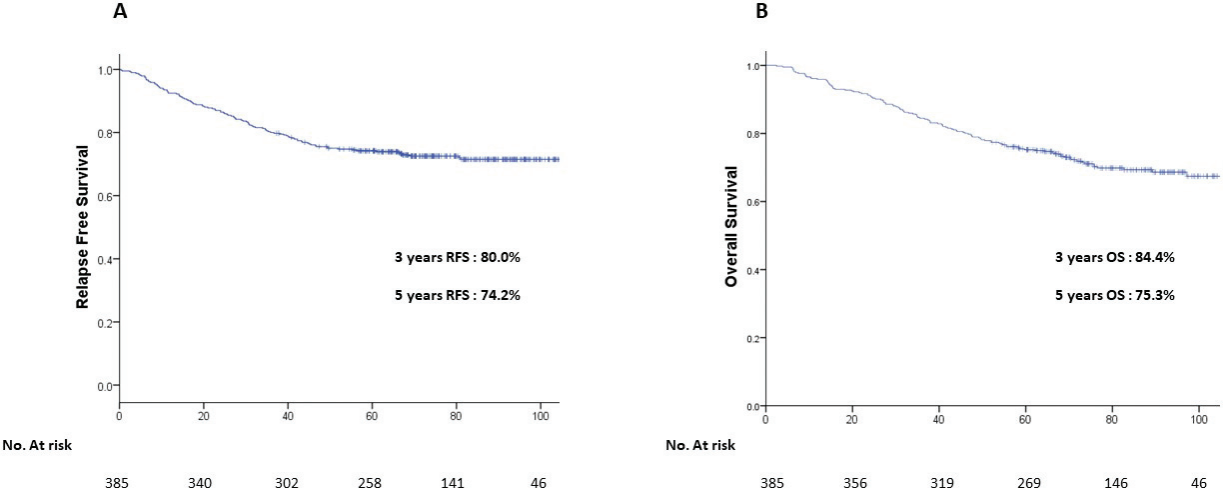
Kaplan–Meier curves of (a): RFS and (b): OS for the entire study cohort of elderly patients with breast cancer.

At a median follow-up of 71.7 months, the 5-year Kaplan–Meier estimates of RFS and OS for the entire study cohort were 74.2% and 75.3% respectively (Figure 1). Five-year LRFS, DDFS, BCSS were found to be 84.8%, 76.1% and 84.5%. The median survival after recurrence was 3 months (range: 1–49 months). Univariate analysis of various patient, disease and treatment-related characteristics (Table 2) identified ‘age at presentation, performance status (KPS), compliance towards treatment, pathological T stage, pathological N stage, presence of LVSI, grade of histology, hormone receptors status (ER/PR/both), HER-2 status and molecular subgrouping (TNBC versus Non-TNBC)’ as important determinants of survival. Patients with age less than 80 years and/or KPS > 80 had significantly higher RFS and OS than patients more than 80 years or KPS < 80. The presence of LVSI, a higher grade was associated with significantly worse survival compared to the absence of LVSI or low-grade histology respectively. Patients with luminal A/B (hormone receptor positivity) breast cancer had the best outcomes, HER-2 rich had intermediate outcomes, while TNBC had the worst outcomes. The presence of comorbidities had no impact on 5-year RFS (*p*: 0.34) and 5-year OS (*p*: 0.56) (Table 2). We did not find any clinically significant survival benefit (5-year RFS (*p*: 0.37) and 5-year OS (*p*: 0.23)) in HER-2-positive patients who received herceptin as compared to those who did not receive.

**Figure 2.**
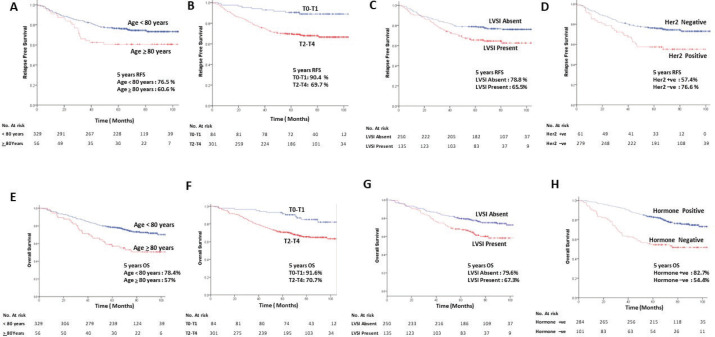
Kaplan–Meier curves of RFS (upper panel) and OS (lower panel) stratified by age (<80 versus >80 years) (a and e); T size (T0-T2 versus T2-T4) (b and f); presence of LVSI (LVSI present versus LVSI absent) (c and g); HER2 receptor status (positive versus negative) (d) and hormonal receptor status (positive versus negative) (h).

On multi-variate analysis, age at presentation, pT size, presence of LVSI, hormone receptors status (ER/PR/Both), HER-2 status emerged as independent predictors of survival (Table 3). The hazards of disease progression and death were significantly reduced for patients with age 70–80 years, pT0/pT1, the absence of LVSI. Similarly, patients with positive hormonal receptor had reduction in the risk of death compared to triple hormone receptornegative patients (Figure 2).

## Discussion

Though the definition of elderly age varies according to the socio-economic status of the country, the cut-off chosen is generally arbitrary. The older patient groups are labelled as ‘elderly’ differently from region to region and it varies contextually. The age for retirement is lower compared to that used in politics or geriatric institutions. Hence there is no particular or typical cut off for defining older people. Chronological age forms an easy and practical way of defining the target population. World Health Organisation defines the elderly as individuals over 65 years [12]. Likewise, the cut-off is 70 years for developed nations and a lower threshold may be chosen for less developed countries. Most of the previously published literature also have taken 70 years as an arbitrary cut off to define the ‘elderly population’ [13]. In this study, at cut-off of 70 years was chosen in the context of breast cancer considering the life expectancy for females in the Asian region is around 70 years [14]. Moreover, the International Society of Geriatric Oncology has published several guidelines for non-breast malignancies using the 70-year definition [15] This cut-off also concurs with the retrospective data showing a sharp increase in geriatric problems after the age of 70 among cancer patients [16].

There is no formal screening program for breast cancer in our country. With the increase in life expectancy in modern times, elderly patients diagnosed with breast cancer should be optimally treated taking into consideration both physical as well as functional status. Moreover, geriatric oncology involves a lot of personalised care as treatment guidelines for elderly cancer patients are not very well defined. Hence treatment strategies that are practiced for the elderly differ compared to the young. The interplay of relative under-treatment and competing risk of mortality from non-cancer causes poses difficulty in drawing firm conclusions of the impact of age on breast cancer outcome. The decreasing life expectance with age, co-existing co-morbidities and physiological changes leading to functional senescence of multiple organs limit the choice of therapies that can be offered to elderly women. This can lead to decisions on sub-standard treatment options based on disease stage as well as reluctance of patient and family to accept evidence-driven therapies that have a small incremental benefit with respect to cancer outcome.

The routinely used performance scales like Eastern Co-operative Oncology Group and KPS cannot be used for the elderly as the impact of co-morbidities is not adequately captured in these scoring systems. Specific indices like Charleston comorbidity index, a comprehensive geriatric assessment is explicitly designed for the elderly [17]. Studies have shown consistently that patients with multiple co-morbidities or higher Charleston comorbidity index have inferior outcomes [18]. In our study, we did not find any significant impact of comorbidities on survival outcomes. The probable reason can be a greater number of patients without comorbidities were presented with higher T size as compared to those with comorbidities and relatively comparable compliance in systemic treatment in both cohorts. A systematic review showed the presence ER positivity, PR positivity and HER2 positivity in 81.1%, 59.3% and 13.4% of the study population, while in our study we found the same in 73.5%, 63.5% and 17.9% of patients respectively [13]. The same review showed the presence of LVSI in 24.4% while in our cohort, 35% of patients had LVSI positive [13]. This explained more adverse prognostic factors in our population resulting in inferior clinical outcomes with 5 years RFS of 75.3% and 5 years OS of 74.2% as compared to the published data.

The majority of our patients underwent mastectomy (85.9%) while only three patients (0.8%) did not undergo surgery because two patients refused the surgery and one was unfit for the surgery. The rates of BCS are higher in the western literature to the tune of 50% when compared to mastectomy in elderly patients [19]. In our population, the higher rates of mastectomy could be explained by the need to avoid further RT, relatively advanced disease, fear of non-compliance due to the risk of toxicity from neoadjuvant therapy and also reduced compliance to follow up. We didn’t find any significant survival difference between patients who underwent mastectomy versus BCS. Concerning the axilla, it is routine to do a sampling followed by axillary clearance in case of positive nodes on sampling at our institute. Sentinel lymph node biopsy is not routinely practiced for clinically node negative axilla due to the involved logistic issues but the procedure of axillary sampling instead of sentinel node biopsy has been validated at our institute [20]. Another less intense approach could be the use of RT and avoiding surgical morbidity for stage I breast cancer, as shown in a population-based analysis of patterns of care in women >65 years [19].

Adjuvant chemotherapy trials mostly exclude elderly women due to questionable tolerance and increased toxicity. Systemic chemotherapy with standard anthracycline and taxane combination remains the standard of care. Majority of the patients in our cohort also received anthracycline-taxane based chemotherapy. However, only 15 patients (21.7%) out of 69 HER2 positive patients received adjuvant trastuzumab in our cohort, the most common reasons being financial constraints and cardiac co-morbidities. Patients with unfavourable cardiac profile did not receive both anthracyclines as well as trastuzumab. A recent study from India showed a substantial increase of HER2-targeted therapy among patients treated at a public hospital in the past decade, likely due to the advent of biosimilars, the use of shorter duration adjuvant regimens [21]. Another study by Cadoo *et al* [22] has shown the efficacy of adjuvant chemotherapy and trastuzumab in elderly patients. However, the patient cohort in this study was age >55 years and <25% of the patients were above 70 years of age.

The role of RT is controversial in elderly women with low-risk early-stage breast cancer. The PRIME (Postoperative Radiotherapy in Minimum Risk Elderly) II and CALGB 9343 trials were primarily conducted for the elderly population and these studies show a modest benefit in local-regional recurrence without any meaningful benefit in OS [23]. Hence patients satisfying the eligibility criteria of such trials can be considered for observation. In our cohort, only 63 (16%) patients had favourable disease that would make them PRIME-eligible.

The predictors of OS in our study were age, pathological T size (pT), presence of LVSI, hormone receptor status. A Surveillance, Epidemiology, and End Results (SEER) 18 database analysis also showed that tumour grade, AJCC Tumour Nodes Metastases (TNM) stage, hormone receptor status, treatment (surgery/RT) were predictors of OS [24]. A National Cancer Database study also showed that in addition to tumour-related characteristics, other factors such as geographic location, financial status and co-morbidities also affect the overall outcomes [25]. The importance of adequate treatment for the elderly is also seen in the Japanese study, where outcomes were directly proportional to the treatment received [26]. In our study compliance towards adequate treatment had an impact on survival in univariate analysis but was not there in multivariate analysis.

### Strength and limitations

This study represents one of the largest mono-institutional series of a cohort of elderly breast cancer patients in India. All patients were treated in a single institute with a uniform protocol. The non-selected population in this cohort is representative of a ‘real-life’ clinical setting, with an almost complete clinical information having a long follow up. The availability of all the possible clinical features including molecular profiling for a whole study cohort provides added value to the study. However, despite the aforesaid strengths, several caveats and limitations remain. The retrospective design of the study makes it susceptible to intrinsic biases that could potentially confound the interpretation of results. A formal geriatric assessment was not done at the time of treatment decision as patients included in this study were treated from 2013 to 2016. Recently, more and more patients are being accrued in the prospective study (CTRI/2021/07/034792) that is evaluating the utility of geriatric assessment tools in the prediction of mortality.

## Conclusion

The audit highlights the underutilisation of breast-conserving therapy and systemic therapy in the elderly. Increasing age, tumour size, presence of LVSI and molecular subtype were found to be strong predictors of outcome. It calls for a systematic evaluation to improve compliance and thus optimal treatment in this cohort of the opulation to improve outcomes. The findings from this study will help to improve the current gaps in the management of breast cancer among the elderly.

## List of abbreviations

OS: Overall survival; RFS: Relapse-free survival; LRFS: Locoregional relapse-free survival; DDFS: Distant disease-free survival; BCSS: Breast cancer-specific survival; pT: Pathological T size; LVSI: Lympho-vascular invasion; KPS: Karnofsky Performance Status Scale; ER: Oestrogen receptor; PR: Progesterone receptor; TNBC: Triple-negative breast cancer; EBC: Early breast cancer; LABC: Locally advanced breast cancer; pN: Pathological N; PNI: Perineural invasion; EIC: Extensive intraductal component; RT: Radiotherapy; BCS: Breast conservative surgery; MRM: Modified radical mastectomy; EBRT: External beam radiotherapy; AC: Adriamycin cyclophosphamide; CAF: Cyclophosphamide adriamycin 5 fluorouracil; CMF: Cyclophosphamide methotrexate 5 fluorouracil; HR: Hazard ratio; CI: Confidence interval.

## Figures and Tables

**Table 1.** Patient, disease characteristics and treatment details of study cohort (N = 385).

Variable		Subgroups		N = 385		Percent
**Patients’ characteristics**						
Gender		Female Male		371 14		96.3% 3.7%
Comorbidity		Present Absent		253 132		65.7% 34.3%
KPS (*n*: 262)		<80 >80		21 241		8.1% 91.9%
Distance from hospital		Within state Outside state		249 136		64.7% 35.3%
Literacy		Literate Illiterate		264 115		68.6% 29.9%
**Disease characteristics**						
Laterality		Right Left Bilateral		178 202 05		46.2% 52.4% 1.4%
Hormone receptor status		ER positive PR positive		283 245		73.5% 63.6%
HER2-nue status		Positive Negative		69 316		17.9% 82.1%
Molecular subtype		Luminal subtype TNBC Her 2 enriched		284 70 31		73.8% 18.2% 8.3%
Clinical stage		EBC LABC		255 130		66% 34%
pT		T0–1 T2–4		83 302		21.6% 78.4%
pN		N0 N+		217 165		56.8% 43.2%
Grade		I–II III		136 249		35.3% 64.7%
Histology		Ductal Lobular Others		359 09 17		93.2% 2.4% 4.4%
LVSI		Present Absent		135 250		35.0% 65.0%
PNI		Present Absent Not reported		33 343 06		8.7% 89.7% 1.6%
EIC		Present Absent Not reported		25 353 04		6.6% 92.3% 2.1%
Type of recurrence (*n*: 49)		Local/Locoregional recurrence Bones metastases only Brain metastases only Lung/liver metastases only Multiple sites metastases		06 08 02 03 30		12% 17% 4% 6% 61%
**Surgical details**						
Primary surgery	Breast conservation Mastectomy No surgery		54 328 03		14.1% 85.9% 0.8%	
Axillary surgery	Level 1–2 clearance Sentinel node Sampling Level 1–3 clearance Not addressed		196 176 08 05		50.9% 45.8% 2.1% 1.2%	
Resection	R0 R+		378 03		99.2% 0.8%	
**Radiation details**						
Adjuvant RT	Yes		194		50.4%	
	No		191		49.6%	
Adjuvant RT to BCS patients (*n*: 54)	Yes No		45 09		83.3% 16.7%	
Adjuvant RT to MRM patients (*n*: 328)	Yes No		149 179		45.4% 54.6%	
Type of RT (*n*: 194)	EBRT		184		94.8%	
	Brachy		10		5.2%	
Boost (*n*: 54)	Yes		18		33.3%	
	No		36		66.7%	
Boost type	Electron		17		94.4%	
	Brachytherapy		1		5.6%	
**Chemotherapy details**						
Neoadjuvant chemotherapy (*n*: 23)	AC		11		47.8%	
	CAF		6		26.0%	
	Taxanes + Trastuzumab		3		13.3%	
	CMF		1		4.3%	
	Taxanes		1		4.3%	
	AC + Taxanes		1		4.3%	
Adjuvant chemotherapy	Anthracyclines alone		23		6%	
	Taxanes alone		34		8.9%	
	Anthracycline and taxanes		29		7.5%	
	CMF		7		1.9%	
	CAF		15		3.9%	
	Others		3		0.7%	
	Not given		274		71.1%	
Maintenance Trastuzumab	Trastuzumab		15/69		21.7%	
Compliance towards treatment	Yes No (Defaulted)		343 42		89.9% 10.1%	

KPS: Karnofsky Performance Status Scale; ER: Estrogen receptor; PR: Progesterone receptor; TNBC: Triple negative breast cancer; EBC: Early breast cancer; LABC: Locally advanced breast cancer; pT: Pathological T; pN: Pathological N; LVSI: Lymphovascular invasion; PNI: Perineural invasion; EIC: Extensive intraductal component; RT: Radiotherapy; BCS: Breast conservative surgery; MRM: Modified radical mastectomy; EBRT: External beam radiotherapy; AC: Adriamycin cyclophosphamide; CAF: Cyclophosphamide adriamycin 5 fluorouracil; CMF: Cyclophosphamide methotrexate 5 fluorouracil

**Table 2.** Univariate analysis of prognostic factors affecting survival outcomes. Clinically significant values are shown in bold.

Prognostic factors		Number of patients (N)	5-year RFS	Log-rank p-value	5-year OS	Log-rank p-value
Age at presentation	<80	329	76.5%	**0.026**	78.4%	**0.001**
	>80	56	60.6%		57.0%	
KPS^a^	<80	21	47.6%	**0.01**	47.6%	**0.026**
	>80	241	75.1%		75.9%	
Comorbidity	Yes	253	72.7%	0.34	74.6%	0.56
	No	132	77.2%		76.4%	
Type of surgery	BCS	54	83.2%	0.11	85.2%	0.081
	Mastectomy	331	72.7%		73.7%	
Compliance to treatment	Yes	343	76.0%	0.009	77.5%	0.013
	No	42	59.5%		56.9%	
pT size	T0–T1	84	90.4%	**<0.001**	91.6%	**0.001**
	T2–T4	301	69.7%		70.7%	
pN status	N0	218	81.6%	**<0.001**	82.0%	**0.001**
	N+	167	64.6%		66.4%	
LVSI	Present	135	65.5%	**0.009**	67.3%	**0.003**
	Absent	250	78.8%		79.6%	
Grade	Grade I–II	136	80.1%	**0.03**	81.6%	**0.008**
	Grade III	249	71.0%		71.8%	
Hormone receptor	Positive	284	81.2%	**<0.001**	82.7%	**<0.001**
	Negative	101	54.5%		54.4%	
HER2 status	Positive	69	76.6%	**0.002**	76.6%	**0.007**
	Negative	316	57.4%		60.6%	
TNBC	Yes	70	58.6%	**<0.001**	58.6%	**0.001**
	No	315	77.7%		79.0%	

RFS: Relapse free survival; OS: Overall survival; KPS: Karnofsky Performance Status Scale; BCS: Breast conservative surgery; pT: Pathological T; pN; Pathological N; LVSI: Lymphovascular invasion; TNBC: Triple negative breast cancer

a Data regarding KPS is available for 262 patients

**Table 3.** Multi-variate analysis of prognostic factors in elderly breast cancer patients. Clinically significant values are shown in bold.

Prognostic factors		RFS			OS		
		HR	95% CI	p-value	HR	95% CI	p-value
Age at presentation	<80 >80 (Ref)	0.55	0.34–0.89	**0.01**	0.40	0.25–0.62	**<0.001**
Compliance to treatment	Yes No (Ref)	0.72	0.42–1.22	0.72	0.62	0.36–1.04	0.07
pT size	T0–T1 T2–T4 (Ref)	0.40	0.20–0.82	**0.01**	0.42	0.22–0.78	**0.006**
pN status	N0 N+ (Ref)	0.66	0.43–1.01	0.05	0.75	0.50–1.12	0.16
LVSI	Present Absent (Ref)	1.49	1.00–2.21	0.04	1.55	1.05–2.27	0.02
Grade	Grade I–II (Ref) Grade III	1.08	0.68–1.72	0.72	0.87	0.55–1.38	0.58
Hormone receptor	Positive Negative (Ref)	0.60	0.31–1.18	0.14	0.37	0.18–0.75	**0.006**
HER2 status^+^	Positive (Ref) Negative	2.25	1.26–4.01	**0.006**	1.32	0.71–2.47	0.37
TNBC	Yes No (Ref)	1.56	0.70–3.49	0.27	0.72	0.31–1.67	0.45

HR: Hazard ratio; CI: Confidence interval; RFS: Relapse free survival; OS: Overall survival; pT: Pathological T; pN; Pathological N; LVSI: Lymphovascular invasion; TNBC: Triple negative breast cancer

## Data Availability

The data that support the findings of this study are available from the corresponding author upon reasonable request.
